# Positive Correlation between nNOS and Stress-Activated Bowel Motility Is Confirmed by In Vivo HiBiT System

**DOI:** 10.3390/cells10051028

**Published:** 2021-04-27

**Authors:** Jeong Pil Han, Jeong Hyeon Lee, Geon Seong Lee, Ok Jae Koo, Su Cheong Yeom

**Affiliations:** 1 Graduate School of International Agricultural Technology and Green, Institute of Green BioScience and Technology, Seoul National University, 1447 Pyeongchang-ro, Daewha, Pyeongchang 25354, Korea; pil1426@snu.ac.kr (J.P.H.); ljhljh7506@snu.ac.kr (J.H.L.); lgs6245@snu.ac.kr (G.S.L.); 2 Toolgen Inc., Gasan Digital-ro, Geumcheon, Seoul 08594, Korea; oj.koo@toolgen.com; 3 WCU Biomodulation Major, Department of Agricultural Biotechnology, Seoul National University, 1 Gwanak-ro, Gwanank, Seoul 08826, Korea

**Keywords:** bowel motility, HiBiT, IBS, NMS, nNOS, stress

## Abstract

Neuronal nitric oxide synthase (nNOS) has various roles as a neurotransmitter. However, studies to date have produced insufficient data to fully support the correlation between nNOS and bowel motility. This study aimed to investigate the correlation between nNOS expression and gastrointestinal (GI) tract motility using a stress-induced neonatal maternal separation (NMS) mouse model. In this study, we generated a genetically modified mouse with the HiBiT sequence knock-in into the *nNOS* gene using CRISPR/Cas9 for analyzing accurate nNOS expression. nNOS expression was measured in the stomach, small intestine, large intestine, adrenal gland, and hypothalamus tissues after establishing the NMS model. The NMS model exhibited a significant increase in nNOS expression in large intestine, adrenal gland, and hypothalamus. Moreover, a significant positive correlation was observed between whole gastrointestinal transit time and the expression level of nNOS. We reasoned that NMS induced chronic stress and consequent nNOS activation in the hypothalamic-pituitary-adrenal (HPA) axis, and led to an excessive increase in intestinal motility in the lower GI tract. These results demonstrated that HiBiT is a sensitive and valuable tool for analyzing in vivo gene activation, and nNOS could be a biomarker of the HPA axis-linked lower intestinal tract dysfunction.

## 1. Introduction

Nitric oxide (NO) is a free radical signaling molecule produced by NO synthases (NOS). There are three isoforms of NOSs as neuronal NOS (nNOS, NOS1), inducible NOS (iNOS, NOS2), and endothelial NOS (eNOS, NOS3). iNOS is expressed in the various cell types and contributes to the inflammation response [1]. eNOS is detected in the endothelial cell and it controls blood pressure and other vasoprotective effects [2]. nNOS is primarily expressed in the central and peripheral neurons and regulates synaptic plasticity, blood pressure, and smooth muscle relaxation via the peripheral nitrergic nerve [3]. Myocardial nNOS overproduction deteriorates cardiac function by a decrease in cardiac contraction [4], whereas chronically lacking nNOS develops gastric dilation and diffuse muscle thickening associated with delayed stomach emptying [5]. Among the three types of NOS, nNOS has a remarkable correlation with external stress [6].

Stress can be caused by various factors that threaten a body’s homeostasis. These reactions affect neurological, endocrine, metabolic, gastrointestinal, and immunological systems [7]. Chronic stress, which can destroy homeostasis and lead to permanent disorder, affects brain function, memory, cognition, learning, immune system functions, and the cardiovascular system [8]. Moreover, stress also deteriorates the gastrointestinal (GI) tract by causing changes in intestinal motility, visceral perception, and the function of the gut barrier [9], and it is an essential factor for the development of irritable bowel syndrome (IBS) [8,10]. IBS is a functional disorder causing irregular bowel movement and an etiology associated with psychological stress and infection [11,12]. Serotonin (5-HT) is well known to play a critical role in stress-related changes in gut motility, visceral sensitivity, and intestinal secretion [13]; also, we previously reported a correlation between nNOS and irritable bowel syndrome [14]. nNOS regulates physiological functions such as learning, memory, neurogenesis, and various human diseases [3,15]. Thus, nNOS is suggested to be essential in regulating stress-induced digestive function, including IBS, but related studies are insufficient.

There are several IBS animal models, such as Trichinella spiralis infection model [16], neonatal maternal separation (NMS) model, and wrap restraint stress model [17,18]. The stress-induced IBS models were well-established, but there is still an unmet need to prove etiology or biomarker. We assumed that nNOS, which is positively associated with the stress response, is an essential regulatory gene for IBS, but there was a limitation on analysis using conventional experimental animals. Recently, novel methods for measuring protein expression with high sensitivity have been developed and applied, such as HiBiT system. HiBiT is luciferase complementation by splitting NanoLuc that consists of 11 amino acid peptides [19]. HiBiT can tag that is fused to the N or C terminus of the protein of interest and detect low intracellular concentration with high conformational stability and low intrinsic affinity [20].

Previously, we reported that nNOS is a novel biomarker of NMS-induced IBS-D type [14]. However, the correlation between nNOS expression and GI tract motility was not analyzed under stress-induced conditions. We reasoned that in vivo HiBiT system would be applicable to identify the correlation between nNOS and stress response. For this, we generated a genetically modified mouse with a 33-base pair of HiBiT sequence knock-in (KI) into the *nNOS* gene C-terminal end using CRISPR/Cas9-mediated single-stranded template repair (SSTR). Next, nNOS expression pattern and correlation with clinical symptoms were analyzed in various organs under an NMS stress-inducing.

## 2. Materials and Methods

### 2.1. Preparation of Single Guide RNA and ssODN

In order to conduct 33 bp HiBiT sequence KI at the C-terminal end of the *nNOS* gene, sgRNA candidate was selected near the stop codon. sgRNA was synthesized using the in vitro RNA synthesis kit (Thermo Fisher Scientific, Waltham, MA, USA) using PCR amplicon. Single strand oligodeoxynucleotide (ssODN) was designed with a HiBiT sequence containing a homology of 40 nt on both sides. It was synthesized using a commercial service (Integrated DNA Technologies, Skokie, IL, USA).

### 2.2. Generation of a Mouse with HiBiT Sequence KI into nNOS Gene C-Terminal End

C57BL/6 mice were purchased from Koatech (PyeongTaek, Korea) and maintained under specific pathogen-free conditions with ad libitum access to feed and water. For superovulation, 5 IU of pregnant mare serum gonadotropin (PMSG) and human chorionic gonadotropin (hCG) were injected at 48 h intervals. Next, the female mice were mated with sperm donors, and embryos were collected from the oviduct on the next day. Embryos with two intact pronuclei were applied to electroporation for gene editing. Briefly, embryos were washed three times with Opti MEM I medium (Invitrogen, Carlsbad, CA, USA). Then, approximately 50 embryos were mixed with electroporation buffer and were transferred into the electrode. The final concentration of electroporation buffer consisted of 200 ng/μL of SpCas9 protein (Toolgen Inc., Seoul, Korea), 400 ng/μL of an ssODN, and 50 ng/μL of each single guide RNA (sgRNA). The electroporation pulse conditions were as follows: 7 cycles of 30 V with 3 ms ON and 97 ms OFF. After washing with the M2 medium (MTI-GlobalStem, Rockville, MD, USA), the embryos were transferred into the oviduct of the recipient female. Genotyping was performed by PCR and Sanger sequencing using toe-clips DNA. The detailed sequences of ssODN, sgRNAs, and primers were listed in Supplementary Table S1. This study was approved by the Institutional Animal Care and Use Committees of Seoul National University (SNU-180315-3-1 and SNU-200221-5) and was conducted as approved guidelines.

### 2.3. Neonatal Maternal Separation

NMS was conducted with minor modifications from previous reports [21,22]. Briefly, the obtained pups were separated from their maternity cages for 3 h (AM 9:00–PM 12:00) daily on postnatal days 3–24, and pups were placed on a warm plate. The control pups remained in their maternity cage.

### 2.4. Protein Preparation

Proteins from each tissue were extracted using RIPA buffer, and quantification was conducted with a BCA protein assay. Serial diluted bovine serum albumin was quantified by measuring absorbance at 570 nm using a spectrophotometer (Tecan, Zurich, Switzerland) and used as a standard. All tissue sample proteins were diluted at 1 µg/µL concentration with distilled water and were subjected to further HiBiT analysis and western blotting.

### 2.5. HiBiT Blotting and HiBiT Lytic Analysis for Detecting nNOS Protein

In HiBiT lytic analysis, protein samples were mixed with a reaction mixture containing HiBiT lytic substrate, LgBiT protein, and HiBiT lytic buffer (Promega, Madison, WI, USA). The volume ratio was 1:1 in the sample protein and reaction mixture. Then, they were incubated at 37 °C for 10 min and measuring luminescence intensity by Cytation 5 (BioTek, Winooski, VT, USA). In HiBiT blotting, proteins were transferred to a nitrocellulose membrane (Thermo Fisher Scientific). Then, the transferred membrane was incubated for one hour at room temperature with LgBiT protein-added Nano-Glo blotting buffer (Promega). Next, the luciferase assay substrate was added into an incubating solution and stayed an additional five minutes at room temperature. After rinsing using Tris-buffered saline solution with 0.05% Tween 20 (TBST), the luminescent signal was detected by Davinci-Chemi (Davinch-K, Seoul, Korea).

### 2.6. Western Blotting

Proteins were transferred to polyvinyl difluoride (PVDF) membranes (EMD Millipore, Billerica, MA, USA) and were incubated with blocking buffer (TBST and 5% *w*/*v* skim milk) for one hour at room temperature. Next, the membrane was incubated with primary antibodies overnight at 4 °C and applied to one hour of incubation with a secondary antibody at room temperature. An enhanced chemiluminescence kit (Abclon, Seoul, Korea) was applied, and the reactive proteins were detected using Davinci-Chemi (Davinch-K). The antibodies used in this study were listed in Supplementary Table S2.

### 2.7. Measurement of NO Concentration

Tissues were homogenized in phosphate-buffered saline, and the supernatants were collected after centrifugation. According to the manufacturer’s instructions, the NO concentrations were measured using an NO plus detection kit (Intron, Seongnam, Korea). The absorbance of NO was measured at 570 nm using a spectrophotometer (Tecan)

### 2.8. Analysis of Whole Gastrointestinal Transit Time

All mice fasted for 18 h before the experiment, and 6% Carmine marker (300 μL; Sigma, St. Louis, MO, USA), including 0.5% methylcellulose solution, was administered via the oral route [13]. The mice were separated individually into translucent cages with white sheets at the bottom. Time from the ingestion until the first observation of the Carmine marker in feces was recorded as the whole gastrointestinal transit time.

### 2.9. Statistical Analysis

Statistical analysis was conducted using unpaired Student’s *t*-test and correlation analysis using GraphPad Prism 8 (GraphPad, San Diego, CA, USA).

## 3. Results

### 3.1. nNOS HiBiT Sequence KI Mouse Was Generated Using CRISPR/Cas9 and ssODN Template

**Figure 1:**
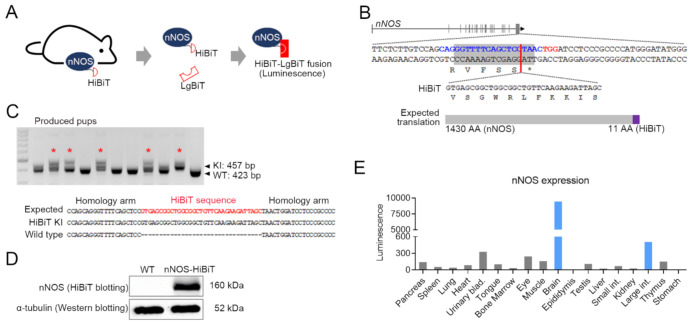
Generation of an nNOS-HiBiT knock-in (KI) mouse and evaluation of HiBiT analysis. (**A**) Brief schematic of nNOS-HiBiT fusion protein for luminescence detection. (**B**) Single guide RNA was designed for 33-base pair of HiBiT sequence KI upstream of the stop codon in the nNOS gene. Blue alphabets: sgRNA binding sequence, Red alphabets: protospacer adjacent motif sequence. (**C**) PCR and Sanger-based genotyping was conducted for obtained pups. Red symbol: KI expected pups, Red alphabet: an expected correct sequence of HiBiT. (**D**) neuronal nitric oxide synthase (nNOS) protein was detected by HiBiT blotting using the nNOS-HiBiT KI and wild type. Western blotting for α-tubulin was conducted to confirm protein integrity. (**E**) nNOS expression was measured by HiBiT lytic analysis with 18 different organs from an nNOS-HiBiT KI mouse. Luminescence value was subtracted by background control.

Conventionally, the target protein detection in organs or cells has been generally measured by western blotting. However, sensitive quantification by western blotting is difficult, and just a rough comparison is possible. Recently, NanoLuc luciferase activity measurement through HiBiT has been reported as an endogenous gene expression analyzing method [23]. We previously reported that the nNOS expresses higher in the interstitial cell of Cajal in the stress-derived IBS mouse model [14], but other organs’ nNOS expression could not be analyzed. In this experiment, the nNOS was selected as the target gene, and we tried to analyze the increase in nNOS expression in stress-derived IBS after HiBiT KI mouse generation (Figure 1A).

The HiBiT system is used by 33 bp of HiBiT sequence KI in the right behind the start codon or before the stop codon to preserve intact endogenous gene expression. In this study, the HiBiT sequence was inserted before stop codon by CRISPR/Cas9-mediated SSTR. To induce SSTR, double-stranded breakage near the target KI site was essential, and there was one sgRNA binding sequence that would induce DSB (−3 bp from PAM) at almost the same site KI. ssODN harboring 33 bp of HiBiT sequence and both homology arms were prepared and co-transfected with Cas9/sgRNA ribonucleoprotein into embryos by electroporation (Figure 1B). A total of 10 pups were obtained, and five pups were proven to have KI (KI efficiency: 50%), and precise KI was confirmed by Sanger sequencing (Figure 1C). Although precise KI would not affect endogenous nNOS protein synthesis level, we utilized the heterozygote nNOS-HiBiT KI mouse in further study to ensure intact nNOS gene expression.

### 3.2. Brain and Large Intestine Exhibited High nNOS Expression

First, we checked whether the HiBiT system works well in in vivo through HiBiT blotting using tissues from nNOS-HiBiT KI mice. As expected, blot through HiBiT was only detected in nNOS-HiBiT KI mice and not in WT (Figure 1D). Subsequently, the nNOS expression level in 18 mouse organs was analyzed using HiBiT lytic detection. The protein expression of nNOS was confirmed to be high in the brain and large intestine (Figure 1E). These results justified further study on the association of nNOS activation with the brain and colon.

### 3.3. NMS Developed a Significant Increase nNOS Expression in the Brain and Large Intestine

**Figure 2:**
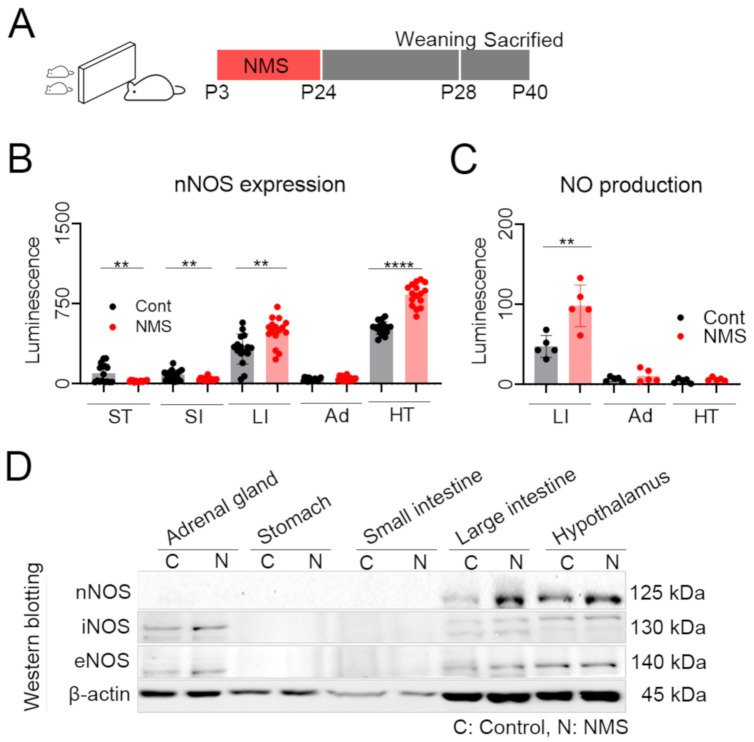
NMS-mediated IBS mouse model and analysis nitric oxide concentration. (**A**) Schematic for inducing NMS-mediated IBS mouse model. Briefly, NMS was conducted for 3 h per day for 21 consecutive days (3–24 days old). Mice were weaned at 28 days old, and sacrificed and sampled at 40 days old. (**B**) nNOS-HiBiT expression was analyzed by HiBiT lytic analysis, and each dot indicates data from the individual animals (control, *n* = 16, NMS, *n* = 16). nNOS-HiBiT expression was displayed as a luminescence intensity and represented as a mean ± SEM. (**C**) Nitric oxide (NO) concentration was analyzed with the large intestine, adrenal, and hypothalamus tissues (control: *n* = 5, NMS: *n* = 5). Statistical analysis was performed using Student’s *t*-test. **: *p* < 0.01 and ****: *p* < 0.0001. ST: Stomach, SI: Small intestine, LI: Large intestine, Ad: Adrenal gland, HT: Hypothalamus. (**D**) NMS was conducted using wild-type mouse, and mice from each group were sacrificed and sampled at 40 days old. Tissue sample proteins from 5 mice with the same group (control vs. NMS) were quantified and merged, and subjected to western blotting to detect iNOS, eNOS, and nNOS. iNOS: inducible NOS, eNOS: endothelial NOS.

We wanted to analyze nNOS expression in the IBS model; thus, the establishment of an IBS model using nNOS-HiBiT KI mice was needed. Various methods for developing mouse IBS models have been reported. Psychological stress has a marked influence on intestinal sensitivity and motility, which can induce some of the IBS’s main symptoms [22]. Based on the recent report for the high connection between the gastrointestinal tract and hypothalamic-pituitary-adrenal (HPA) axis [24], establishing a chronic stress-inducing IBS model was determined (Figure 2A). nNOS expression was measured in the stomach, small intestine, large intestine, adrenal gland, and hypothalamus tissues after establishing the NMS-mediated IBS model. The NMS model exhibited significant changes in nNOS expression compared to the control group. The NMS developed lower nNOS expression in the upper GI tract (stomach and small intestine) but it was significantly higher in the lower GI tract (colon) (Figure 2B).

Because nNOS expression was increased, NO concentration needed to be measured in the large intestine, adrenal gland, and hypothalamus (Figure 2C). As with the nNOS expression pattern, NO concentration was increased in the large intestine, but there was no detectable change in the adrenal gland and hypothalamus. There are three types of NO, so the protein expression of other NOs should be examined. Thus, we analyzed the iNOS, eNOS, and nNOS in each tissue and observed that only nNOS increased in the large intestine and hypothalamus. However, there were no significant changes in iNOS and eNOS.

### 3.4. Whole Gastrointestinal Transit Time Was Positively Correlated with Expression Level of nNOS in the Hypothalamus-Adrenal Gland-Large Intestinal Axis

**Figure 3:**
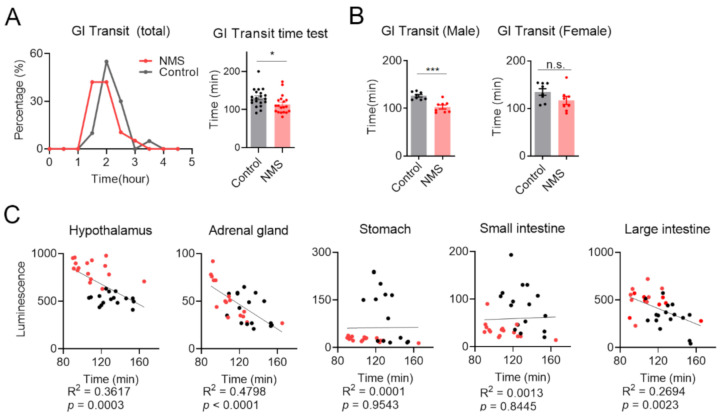
Positive correlation between nNOS expression and whole gastrointestinal transit time. (**A**) Whole gastrointestinal transit time was measured in NMS and control mice. The time of the first observation of carmine-stained feces was designated transit time. Transit time was analyzed by grouping for 30 min (left graph) and compared using individual transit times (right graph) (control, *n* = 16, NMS, *n* = 16). (**B**) Transit time from male and female mice was analyzed (male and female: control, *n* = 8, NMS, *n* = 8). Each dot indicates data from the individual animals, and data were represented as a mean ± SEM. Statistical analysis was performed using Student’s *t*-test. n.s.: not significant, *: *p* < 0.05, and ***: *p* < 0.001. (**C**) Correlation analysis for nNOS expression and whole gastrointestinal transit time on several organs was conducted. Each dot indicates data from individual mice. Black dot: data from control mice, Red dot: data from NMS mice. Statistical analysis was performed using Pearson R and Student’s two-tailed *t*-test.

The whole gastrointestinal transit time test is a tool that assesses intestinal motility and can be used to evaluate diarrheal type IBS (IBS-D). When stress causes too much intestinal muscular relaxation, the gastrointestinal transit time is reduced and faster expelled of the intestinal contents [25]. As expected, the NMS mice presented a shorter whole gastrointestinal transit time than control (Figure 3A). In humans, IBS differs in the severity of clinical symptoms according to gender. Constipating IBS (IBS-C) is observed more frequently in women, while IBS-D is more often observed in men [26]. Both sex mice seemed to induce IBS-D, but male mice exhibited a more significant difference in bowel motility under NMS (Figure 3B). The more pronounced IBS-D in male mice appeared similar to human clinical manifestations and our previous report [14].

We reasoned that NMS induced chronic stress and consequent nNOS activation in the HPA axis, and led to an excessive increase in intestinal motility in the lower GI tract. As expected, a significant correlation was observed between expression level of nNOS and whole gastrointestinal transit time (Figure 3C). When nNOS was activated, the whole gastrointestinal transit time decreased proportionally. These results suggest that nNOS is positively associated with diarrhea symptoms in NMS-mediated stressful situations. Since there was no characteristic finding in the upper digestive tract, long-term stress such as NMS primarily seemed to affect the lower digestive tract’s motility.

## 4. Discussion

IBS is a representative stress-related functional bowel disease with a prevalence of 10–20% [27] and is considered multifactorial, but the exact pathophysiological etiology is unclear. We already have reported that IBS-D increases nNOS expression in the interstitial cell of Cajal [14]. In this study, we tried to confirm the association of nNOS with stress and bowel motility using the in vivo detecting system. After producing a reporter mouse with 33 bp HiBiT sequence KI in C-terminal end of *nNOS* gene, we induced an NMS experimental IBS model. In particular, we identified the positive correlation between expression level of nNOS and bowel motility in the NMS-induced IBS-D model. These results demonstrated that HiBiT is a sensitive and valuable tool for in vivo gene expression analysis and nNOS is a biomarker of the bowel motility of the HPA axis-linked lower intestinal tract.

Regulation of gene expression is the process by which the body responds to internal and external stimuli. We confirmed that the amount of nNOS was positively correlated with clinical diarrhea symptoms using the in vivo HiBiT lytic analysis. HiBiT is a tool that can detect luciferase intensity by two cleaved luciferase protein fusions. Moreover, HiBiT can ensure high sensitivity and accurate quantification, which is a characteristic that is difficult to conduct with conventional experimental methods such as western blotting. Studies using the HiBiT system at the cellular level have been reported [19,20], but there have been no cases in which it has been applied to animal models. As demonstrated in this study, in vivo HiBiT lytic analysis is easy and has the advantage of enabling quantification. Therefore, it is expected that it can be applied to various gene expression analysis studies.

There are several methods for establishing IBS animal models, such as central nervous stimulation-induced, chemical stimulation-induced, mechanical stimulation-induced, and bacterial or parasite infection-induced [22]. Central nervous stimulation induces brain activity change and further affects the gut’s function through the brain-gut axis. The IBS animal model induced in a similar way to human patients is a central stimulation-induced model such as water avoidance, restraint stress, and neonatal maternal separation. NO production was increased in plasma and rectal tissues of IBS patients [28,29], and NOS analysis of the distal colon of the IBS rat model induced by NMS demonstrated a significant increase in nNOS. However, there was no change in iNOS and eNOS [30]. Our study also exhibited that NMS induced significantly high nNOS expression in the hypothalamus-adrenal-Large intestine axis but did not show significant changes in iNOS and eNOS. This result suggests that mainly nNOS among the NOSs correlates with stress-mediated lower intestinal dysfunction.

In the GI tract, nNOS has various roles as a neurotransmitter. The overproduction of NO by the peripheral nitrergic nervous nNOS might cause gastroesophageal reflux disease [31]. Similarly, this study also presented a significant increase in nNOS expression in the large intestine and indicated that nNOS could be considered a therapeutic target for stress-induced IBS. Although nNOS is proposed as a therapeutic candidate in the NMS-derived IBS-D, there is still a need for confirming enhancing clinical IBS-D symptoms by regulating the nNOS pathway in vivo model. Moreover, there were several types of stress-induced IBS models, so the link between nNOS and HPA axis should be re-evaluated with a different platform.

In summary, we demonstrated a sensitive tool for analyzing gene function. By applying the HiBiT system, research on targets that have been difficult to analyze in conventional methods can be carried out. In addition, the NMS model showed increasing nNOS expression in the large intestine, adrenal gland, and hypothalamus and was positively associated with whole gastrointestinal transit time. Thus, we suggest nNOS as a biomarker and candidate therapeutic regulating pathway for stress-mediated bowel dysfunction.

## Data Availability

All relevant data are included within the manuscript and supplementary file. The raw data are available on request from the corresponding author.

## Supplementary Materials

The following are available online at https://www.mdpi.com/article/10.3390/cells10051028/s1, Table S1: The detailed sequence information of primer, sgRNA and ssODN, Table S2: List of antibodies used in this study.
