# Supplementary material for: Positive Correlation between nNOS and Stress-Activated Bowel Motility Is Confirmed by In Vivo HiBiT System
Source: Cells. 2021 Apr 27;10(5):1028. doi: 10.3390/cells10051028 (PMC8145384; doi:10.3390/cells10051028)
Supplement: Supplementary file 1 [file cells-10-01028-s001.zip › cells-1192617-SI.pdf]

Supplementary information for

Positive correlation between nNOS and stress-activated bowel motility is confirmed by *in vivo* HiBiT system

Short title: IBS, nNOS, and HiBiT

Authors

Jeong Pil Han[1], Jeong Hyeon Lee[1], Geon Seong Lee[1], Ok Jae Koo[2], Su Cheong Yeom[1,3*]

Affiliation

[1]Graduate School of International Agricultural Technology and Green and Institute of Green BioScience and Technology, Seoul National University, 1447 Pyeongchang-Ro, Daewha, Pyeongchang, Gangwon 25354, Korea

[2]Toolgen Inc., Gasan Digital-Ro, Geumcheon, 08594, Seoul, Korea

[3]WCU Biomodulation Major, Department of Agricultural Biotechnology, Seoul National University, 1 Gwanak-ro, Gwanank, Seoul 08826, Korea

Corresponding authors

Su Cheong Yeom, DVM, Ph.D., Associate professor, Graduate School of International Agricultural Technology, Seoul National University, 1447 Pyeongchang-Ro, Daewha, Pyeongchang, Gangwon 25354, Korea

Tel: 82-33-339-5750, Fax: 82-33-339-5762, E-mail: scyeom@snu.ac.kr

This file included

1. The detailed sequence information of primer, sgRNA, and ssODN
2. List of antibodies used in this study

Supplementary Table S1. The detailed sequence of primers, ssODN, and sgRNAs

Gene		sgRNA binding sequence
nNOS	Primer F	5'-TGGAGAGTTAGGGGCAGT-3'
	Primer R	5'-GGGTTGGGGTTGAGAGAAG-3'
	ssODN	CCCTGACCCCCGAAGAAAGCTAAGGAACCTGGTTTCCACGCCACTAACACCTTCTCTTGTCCAGCAGGGTTTTCAGCTCCGTGAGCGGCTGGCGGCTGTTCAAGAAGATTAGCTAACTGGATCCTCCCGCCCCATGGGATATGGGGTGGCTGCCCTGAGTGCCCACGCGAGGGCGGCCGCAAGTTGACTAAAC
	sgRNA 1	CAGGGTTTTCAGCTCCTAAC

Supplementary Table S2. List of antibodies used in this study

	Target	Clone	Host	Dilution	Company
Primary antibody	iNOS	Polyclonal	Rabbit	1:500 (WB)	Biorbyt
	eNOS	Polyclonal	Rabbit	1:500 (WB)	Biorbyt
	nNOS	Polyclonal	Rabbit	1:500 (WB)	Biorbyt
	α-tubulin	Polyclonal	Rabbit	1:3000 (WB)	Cell Signaling
	β-actin	D6A8	Rabbit	1:3000 (WB)	Cell Signaling
Secondary antibody	Rabbit (HRP)		Goat	1:2000	Santa cruz
